# Osmolyte-Induced Folding and Stability of Proteins: Concepts and Characterization

**DOI:** 10.22037/ijpr.2020.112621.13857

**Published:** 2019

**Authors:** Somayeh Mojtabavi, Nasrin Samadi, Mohammad Ali Faramarzi

**Affiliations:** a *Department of Pharmaceutical Biotechnology, Faculty of Pharmacy, Tehran University of Medical Sciences, Tehran, Iran. *; b *Department of Drug and Food Control, Faculty of Pharmacy, Tehran University of Medical Sciences, Tehran, Iran.*

**Keywords:** Osmolyte, Preferential hydration, Protein structure, Protein stability, Folding state

## Abstract

It is well-known that the typical protein’s three-dimensional structure is relatively unstable in harsh conditions. A practical approach to maintain the folded state and thus improve the stability and activity of proteins in unusual circumstances is to directly apply stabilizing substances such as osmolytes to the protein-containing solutions. Osmolytes as natural occurring organic molecules typically called “compatible” solutes, based on the concept that they do not perturb cellular components. However, urea and guanidine hydrochloride (GuHCl) as denaturing osmolytes destabilize many macromolecular structures and inhibit functions. Several studies have been so far performed to explain the actual interaction of an osmolyte with a protein. The present review is aimed to achieve a collective knowledge of the progress arise in the field of osmolyte-protein interactions. The following is also an overview of the main techniques to measure protein stability in the presence of osmolytes.

## Introduction

Proteins are comprised of a biopolymer of amino acids in which linear arrays of their monomers fold up and form a compact 3D structure (1). As a complicated system, proteins are present in various conformations in their folded states. The thermodynamically stable conformation of a protein is based on the hydrophilic and hydrophobic properties of amino acid sequences, chains of amino acids, and interactions that they create with each other and surrounding solution (2–4). Hostile environments featured with stresses as extreme temperature, desiccation, high salt level, severe pH, dehydration, and even exposure to denaturing concentrations of urea may destabilize a protein (5). When destabilized, protein may lose secondary and/or tertiary structures. Moreover, some proteins stand unfolded structures in nature even under native conditions (6).

To get higher stability of biological macromolecules in solid and liquid dosage forms, various stabilizing techniques have been so far developed. For stabilization, it is important to decrease the molecular motions and reduce unfavorable conformational transitions in return. Suitable stabilization methods such as freezing, cooling, freeze-drying, and desiccation alter the thermodynamic state of a protein throughout affecting its surrounding solution (7–10). There are different advantages in each technique depending on properties, applications, or shelf life of the adopted drugs (10). To improve the stability of a protein, stabilizer substances and osmolytes such as sugars, polyols, and amino acids can be supplemented to the formulation (11–14). The key features of such stabilizers are their capability to change the protein structure and the motions of water molecules, which lead to higher stability. Studying and comprehending the effect of these compounds on structure, folding, and function of a protein has taken about half a century. Several articles have specifically focused on osmolyte-protein interactions (12–15). The present review is aimed to give a collective knowledge of some aspects of progress made on osmolyte-protein interactions. Among the variety of features discussed here, the influences of osmolytes on protein folding landscape, thermodynamic mechanism, and enzymatic kinetic parameters are also investigated in brief (15–16).

*Protein stability*

A protein, to carry out a specific biological function, has to adopt a unique native structure in aqueous solution under physiological conditions (17). The stability of protein samples is known as one of the major concerns in many applications, e.g. the pharmaceutical industry, general biochemical studies, and particularly in the field of structural biology. The native structure of a protein is usually very sensitive to any change in its environmental properties such as pressure, temperature, salinity, humidity, and so forth (17–19). Unfortunately, protein possesses chemical and physical properties presenting unique difficulties in purification, storage, and delivery.

Protein degradation pathways are usually divided into chemical and physical classes. Chemical degradation is defined as any process involves modification of a protein via formation or cleavage of covalent bonds or generating new chemical entities (20–21). Conversely, physical instabilities refer to any change in higher-order (secondary or over) structure of a protein, without alteration of its chemical composition. Four procedures commonly involve in physical instability including denaturation, aggregation, precipitation, and adsorption (19–23). The more commonly observed chemical degradation processes are listed in Table 1. As of late, it has been demonstrated that physical and chemical instabilities of proteins are interrelated in many systems (24–25). Similarly, there are examples of certain procedures of chemical degradation (e.g. deamidation) that make a protein more likely to form and aggregate fibrils. This is particularly essential for the purposes of debate and mechanism elucidation to differentiate physical and chemical instabilities. Manning *et al*. proposed some strategies for improving the stability of proteins. The most common of these strategies and advances are summarized in Table 2. (17, 24 –26). Nevertheless, protein stability remains one of the most important hurdles for the development of new the function.

*Osmolytes*

**Figure 1:**
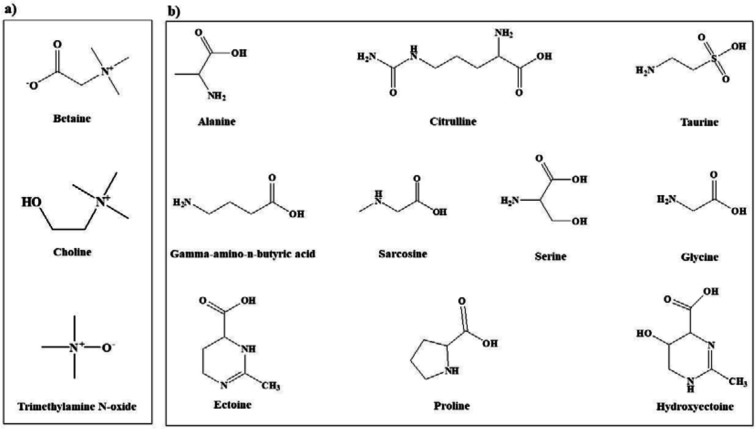
Chemical structures of the most common osmolytes belonging to (a) methylamine and (b) amino acid classes

**Figure 2:**
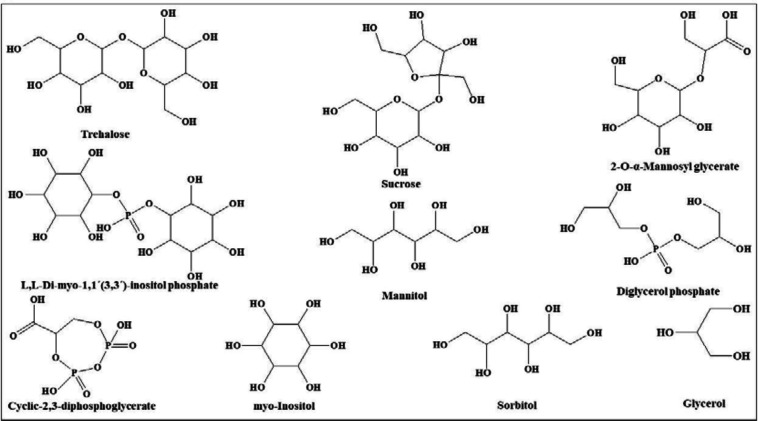
Chemical structures of osmolytes classified in polyols

**Figure 3:**
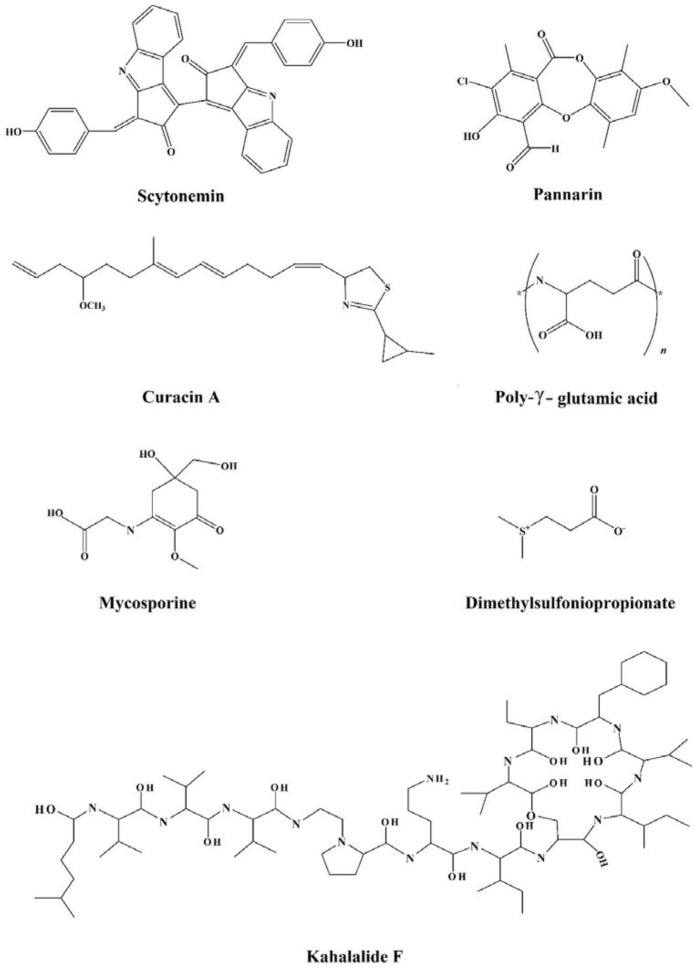
Chemical structures of the miscellaneous group of osmolytes

In view of the large variability of environmental conditions, cells, or whole organisms are exposed to potentially harmful fluctuations in pressure, pH, ion concentrations, temperature, etc. but it is amazing how their sensitive macromolecules react to environmental changes (27–28). All organisms are virtually equipped with small organic compounds termed osmolytes to protect their protein and enzymes (27). The root words of osmolyte are ancient Greek, including osm- meaning “push” or “thrust” and -lytós meaning “soluble” or “dissolve”. Osmolyte commonly referred to the compound affecting on spontaneous movement of solvent molecules into a region of higher solute concentration. An osmolyte is soluble in solution within a cell, or in the surrounding fluid. It plays a role in maintaining cell volume and fluid balance. Osmolyte as a low-molecular weight solute accumulates *in-vivo* under stress conditions and influences on the stability of proteins in living cells (28). The effect of naturally occurring osmolytes on protein conformational stability has been studied for a long time. Protein stability and associations depend on the steric, van der Waals, hydrophobic, and electrostatic interactions of the protein with itself and all solution components. Therefore, modification of the surrounding solution is one of the best approaches to increase the stability and activity of proteins (27, 29–31). The most usual classification of osmolytes is based on their chemical structure such as free amino acids (e.g., proline and glycine) and their derivatives, methylamines (e.g., sarcosine, trimethylamine N-oxide, and betaine), sugars (e.g. sucrose, and trehalose), polyols (e.g. glycerol, glucosylglycerol mannosylglycerol, and arabitol), and ectoines (ectoine and β-hydroxyectoine) (Figures 1−3). 

**Figure 4:**
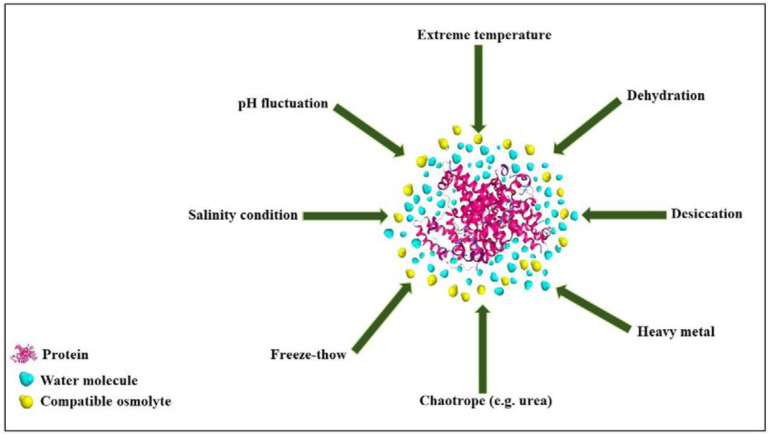
Schematic representation of the preferential exclusion of a compatible osmolyte on protein under stress conditions

However, osmolytes belong to a single chemical class may have not similar effects on protein stability and functional activities vice versa; osmolytes of different classes may have similar effects on proteins (32–33). Other categories have been proposed, in which osmolytes are classified according to their activities. In a well-known classification, osmolytes are categorized according to their denaturing attributes (e.g., urea, guanidinium chloride, lysine), or osmo-protective properties (the majority of the other osmolytes). Protecting osmolytes, known as compatible osmolytes, bias the protein’s structure toward the folded state without unfavorable interactions with proteins or perturbing their structure and function (Figure 4). Table 3 illustrates the stabilization effects of various classes of compatible osmolytes on proteins. Compatible osmolytes can induce intrinsically disordered proteins to fold into the native and functional forms. It has been shown that incubation with the osmolytes causes the intrinsically disordered activation domain of a protein to fold into a form that could bind strongly to a specific receptor (12). Urea and guanidine hydrochloride (GuHCl) belong to the class of denaturing organic or non-protecting osmolytes tending the folding transition toward the unfolded state. It seems that the stabilizing or destabilizing property of osmolytes is universal and independent of the chemical characteristics of a protein (29). Despite the denaturing effects of GuHCl and urea, they are interestingly accumulated in high concentrations in several species (e.g. marine elasmobranchs, mammalian kidneys, and amphibians) (32, 34)*.*

*The influence of osmolytes on molecular interactions*

Understanding the mechanism of action of an osmolyte is crucial to maintain the stability and functionality of proteins *in-vivo*. Despite extensive research, there is no single view on the exact mechanism of action of an osmolyte. Some believe that osmolytes interact directly with protein backbone peptides or amino acid side chains; however, it should be noted that osmolytes do not significantly change the structures of native proteins (17, 29). To date, most published researches have attempted to describe the influence of compatible osmolytes on protein tertiary structure and stability. These osmolytes affect protein folds through a mechanism that targets the amide backbone of the protein, although the exact mechanism remains controversial (38–41). Stabilizing osmolytes are preferentially excluded from protein surfaces, driven by a thermodynamic distaste for the protein backbone. Urea and GuHCl, as the denaturing osmolytes, destabilizes the structure of proteins via favorable interactions with the backbone (66–67).

The hydration structure of osmolytes is affected by high pressure, high/low temperatures, and additions of salts to osmolyte-water systems. It means that the hydration shells are stable in solutions under harsh environmental conditions. Stable hydration shell may act as a defensive barrier to keep proteins from subsequent unfolding, denaturation, aggregation, and retention of functional activity of proteins. For example, the osmolyte hydration shell plays a prevention role against inorganic ions to penetrate to the protein surface, destroy the hydration shell, and denature the protein (28, 68). Inorganic ions may bind to osmolytes in a cooperative manner. As a result, this cooperative binding protects the protein from salting-out that helps in turn the structure stabilization. Therefore, the formation of the stable hydration shell prevents direct interactions of cations and anions to the protein (28, 68).

*Osmolyte-protein interaction*

It is assumed that the solubility and stability of a protein in osmolyte-containing solutions are functions of protein solubility in water and osmolytes (65). Recently, Kirkwood-Buff”s theory was considered for the determination of hydration and osmolation (osmolytes solvation) of proteins for all the classes of osmolytes. It is believed that the hydration of a protein side-chains caused by the presence of osmolytes is much more variable in the different osmolytes solutions. Therefore, osmolytes can be classified according to their solvation behavior on peptide units (65–67). Methylamines (e.g. TMAO, sarcosine, and betaine) are strongly excluded from the protein surface showing few changes in the hydration of the molecule (37–39). In the presence of amino acids such as proline and polyols, as osmolytes, the amount of water is also excluded from the protein surface and exerts their effects differently rather than methylamines. These osmolytes unfavorably interact with the protein and excessively hydrate around it (52–54). Urea, as a denaturing osmolyte, obeys the classical solvent exchange mechanism in which the preferential interaction with the peptide unit excludes water (65–67).

*Osmolyte-induced changes in protein conformational equilibrium*

The effect of osmolytes on the equilibrium protein-folding reaction (Native state (N)↔Unfolded state (U)) has been used for understanding their mechanism of action. This may also show the stabilizing effects on proteins within the range of the least and most effective osmolytes (69–70). Through the non-perturbing effects on the interactions between charged protein subunits, osmolytes (such as glycine, alanine, and betaine) are compatible with protein surface interactions. These molecules are mostly zwitterions or uncharged so that adding a methyl side chain to osmolytes decreases the stabilization of a protein (35–38). Also, the trimethylation of some osmolytes (e.g. betaine) drops down stabilization even more. As understood by molecular dynamics, the dipole of zwitterionic osmolytes (e.g. betaine, proline, sarcosine, and TMAO) is restricted from electrostatic interaction with dipoles by hydrophobic substituents (35, 70). This behavior can be explained by the benign effect of these osmolytes on protein-protein interactions. Generally, to improve stability, specificity, orientation, and the rate of protein-partner recognition, the interfaces of proteins are charge optimized (65–66). Thus, it is clear that nature selects those osmolytes in the pool of available organic molecules that are preferentially excluded from protein surfaces and at the same time cannot interact with surface charge. This means that they are non-perturbing to protein-protein interactions (68, 70). Electrostatic interactions between biomolecular surfaces are a fundamental component of cellular structure-function, and integrity. Osmolytes are expected to affect binding reactions as well as the conformational equilibria (71, 72). Osmotic stress endangers cells that lose cytoplasmic water and experience an increase in macromolecular crowding, which in turn decreases protein stability and can lead to deleterious aggregation of biopolymers. Therefore, molecules that screen repulsive electrostatic interactions or promote protein association, such as the non-osmolytes evaluated (e.g. citrate, acetate, and spearmint) accumulate in cell (68, 70).

Osmolytes do not delay the step that leads to aggregation but rather decrease the accumulation of aggregation competent partially unfolded states. However, at high osmolyte concentrations, compact and off-pathway intermediates might accumulate causing the drawback of delaying protein folding. Conformational compaction induced by which increases the folding rate, might also lead to non-native interactions that have to be disrupted before reaching the native state (29, 72). Some studies discussed the effect of an osmolyte on its own binding to a protein. Aggregation could be also considered a solvation phenomenon that involves two protein states that merely differ in their solvation characteristics (29, 73, 74). In contrast, Fedotova (68) believed that an increase of osmolyte concentration up to the concentration close to saturation significantly leads to dehydration and H-bonding weakening of osmolytes but without important changes in the size of their hydration shells. Therefore, at a high concentration of osmolytes, water molecules are replaced by osmolyte molecules and hydration shell is retained sizeable. However, even in these crowded conditions the osmolytes have rather large hydration numbers. It is suggested that even at a large osmolyte concentration in the cell, osmolytes and proteins and are distinct by a water layer, and do not have direct interactions (28, 68, 70).

*Osmolytes and water interaction*

There is no doubt that the addition of osmolytes somehow alters water structure. Many methods were used to define water structure, for example, in terms of numbers of hydrogen bonds and their length, or the average density of water molecules at various distances from other water molecules. Each of these different definitions has its own purpose and is valuable in various contexts (29, 70–72). In the context of the thermodynamics, there is a direct relationship between preferential interaction of an osmolyte and a protein for its stabilization. Comparing with the native state, exclusion of stabilizing osmolytes from the protein-unfolded state is stronger. Thus, the extent to which bulk water functions as a better solvent of osmolytes comparing with water in the vicinity of a protein determines this type of influence of osmolytes (72–74). Molecular Dynamic Simulation (MDS) studies on osmolytes indicate that trimethylamine N-oxide (TMAO), which is methylamine, trivially increases the number of water-water hydrogen bonds. Therefore, TMAO eliminates the ability of water molecules to compete for intramolecular hydrogen bonds through its structure-making action. In contrast to TMAO, polyols (such as glycerol, xylitol, mannitol, and sorbitol) can intervene in water structure and decrease water ordering (34, 72). Notably, there is a correlation between the extent of hydrogen bond loss and water disordering and proportion to the number of polyol hydroxyl groups, osmolyte internal hydrogen bonds count, and specific property of an isomer (52–54). Therefore, the results of different osmolytes exhibit that their effect on water structure is completely different. So, the formation of more distorted hydrogen bonds between water and an osmolyte cannot be considered as the main reason for the native protein structural stabilization and exclusiveness from the surface of a protein (75). It has been demonstrated that some osmolytes (such as trehalose) without excluding from the protein’s surface, binds to the native state of a protein and make it stabilized (76). Studies convincingly show that there is a little correlation between osmolytes’ stabilizing effect and their impact on water structuring in aqueous solutions (34, 74–76).

*Effects of osmolytes on internal dynamics/ native state flexibility*

Several studies have explained the compatibility paradigm of osmolytes in the face of protein stability, the midpoint of denaturation (melting temperature (*T*_m_) or heat capacity (*C*_m_)), enzyme kinetic parameters (the turnover number (*k*_cat_) and Michaelis–Menten constant (*K*_m_)), the free energy (Δ*G*), and relation thereof (77). Thermodynamically, Δ*G* between the native and denatured states can use to determine osmolyte-induced protein folding. It is recently reported that free-energies of side chain transfer from water to osmolytes can be predicted by achieving solvent dependent cooperative protein native/unfolding free-energy (in terms of *m* values) (78). Based on many pieces of research, polyols and amino acids, or their derivatives show no considerable impact on *k*_cat_ and the free energy of the unfolding state of proteins. Methylamines amplify both *k*_cat_ and ∆*G*^0^_u_ and decrease the Michaelis–Menten constant (*K*_m_). Other groups of osmolytes, including sugars, decrease both *K*_m _and *k*_ cat_ but increase ∆*G*^0^_u _(77–80). However, it is important to note that compatibility also depends on the nature of the applied protein. Indeed, these results are in contrast to the stabilization afforded by excluded solutes. Osmolytes are excluded from the vicinity of the protein surface; therefore, no direct interaction is found between the protein and osmolytes. Based on this theory, osmolytes are expected to have no effect on *K*_m _and *k*_cat_. Despite differences in the interpretation of the results obtained from various techniques, there is no direct relationship between thermodynamic stability and activity of enzymes and proteins in the presence of osmolytes (78, 81). Nevertheless, the effect of osmolytes on protein dynamics cannot be discounted. It is suggested that various classes of osmolytes have different consequences on the native structure ensemble. Although compatible osmolytes are well-known to stabilize proteins, it has not ignored the use of protein destabilizing or non-compatible osmolytes to act as efficient osmo-protectant. Chaotropic substances such as urea disrupt non-covalently responsible for the structure of proteins and influence enzyme kinetic parameters such as *V*_max_ (maximal velocity), *K*_m_ and alter the *C*_m_. Arginine, lysine, and histidine are also known as common non-compatible osmolytes, decrease both *T*_m_ and the Gibbs free energy change on denaturation of proteins at physiological conditions (78–81). Arginine is found to destabilize protein due to preferential binding to proteins. It is speculated that non-compatible osmolytes act as ligands to many intracellular proteins and directly modulated the functional activity of proteins (35, 79–82).

*Structural thermodynamics of protein preferential solvation*

**Figure 5:**
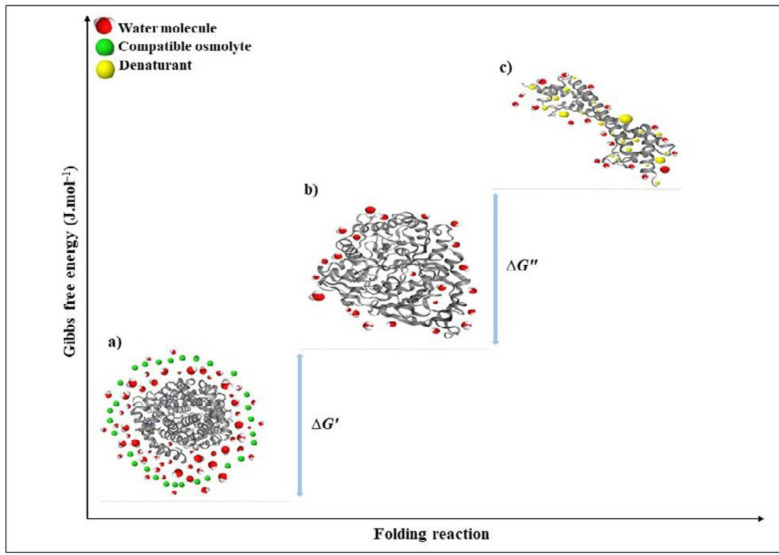
The mechanism and thermodynamic effects of stabilization and destabilization of a protein in the presence of osmo-protectants and denaturants

Proteins are naturally dynamic molecules with marginal stability and a free energy difference, in the folded and denatured state. Undoubtedly, N↔U is important from a biochemical studies point of view; however, usually the denaturation process is not a chemical reaction since no covalent bonds are made or broken (83). Thermodynamic pull drives a protein to its 3D folded structure depending on the amino acid sequence and the surrounding environment. Enthalpy and entropy as key factors contribute to thermodynamic pull between the folded and unfolded states (∆*G*) (83–86). Enthalpy change (Δ*H*) between the native and unfolded state is generally contributed by the non-covalent interactions in the polypeptide chain, such as van der Waals, electrostatic, hydrophobic interactions, hydrogen bonding, and also form covalent disulfide bonds (73). These interactions are found to a greater extent in the native state than the unfolded states. The disorder in the unfolded state of a protein is very high in comparison to the native form. However, conformational entropy difference contributes to the total energy between folded and unfolded states in the opposite direction to the enthalpy influence. Interactions of the polypeptide chains with each other and the surrounding solvent contribute the protein stabilization energy for the folded state (83–86). The force generated from hydrogen bonding between water molecules buries hydrophobic parts of a protein. The hydrophobic effect reduces conformational freedom of water around the protein hydrophobic side chains that cause a decrease in entropy of the system (83, 85). Therefore, the protein backbone collapses into a dense globule and results in the stability of a compact and low entropic form, which is the native state (83–86). In the unfolded state, the hydrophobic effect is more dominant with more exposed hydrophobic groups. Taken together, the native state of a protein has greater entropy due to the hydrophobic effect than the denatured form, while conformational entropy is opposite. Ultimately, the energy difference between folded and unfolded structures (∆*G*) is very small, indicating the borderline stability of the protein (Figure 5) (81–82).

*Structural characteristics of the folding state of a protein Circular Dichroism (CD)*

About five decades ago, machines first became available that were capable of measuring below 250 nm, where the protein backbone amides absorb light. Since then, CD been become more valuable as a rapid technique to study interactions and the folding of proteins. Amide polypeptide bonds aligned in regular arrays such as β-sheets or α-helices show characteristic spectra. Usually, proteins with high contents of α-helices and β-sheets have respectively characteristic bands at 222 or 208 nm and 210 or 220 nm (86–89). The addition of denaturants or stabilizing agents, such as osmolytes often changes protein’s CD spectra. Therefore, today CD is used to investigate the effects of osmolytes and denaturants on protein folding, or to determine ligand-binding constants. In general, compatible osmolytes *in-vitro* enhance the stability of many proteins without substantial changes in their functions. It has been suggested that osmo-protectants have a property forcing proteins to fold, and this general solvophobic property has been termed the osmophobic effect. Bolen *et al*. (85) proposed that osmolytes unfavorably interact with the peptide backbone and exert mainly their stabilizing effects on proteins. A hypothesis that emerges from this idea is that osmolytes act as structure-inducing agents to induce helical structure in otherwise unfolded polypeptides. As predicted by the osmophobic effect hypothesis, many osmolytes such as sucrose and TMAO induce helix formation (84, 87). Nevertheless, urea is known to induce helix unfolding in a peptide backbone. Therefore, nonprotecting and protecting osmolytes are identical in their ability to unfold or refold proteins’ α-helix, respectively (88–90). Many studies have summarized the use of CD spectroscopy to find the free energy of folding proteins as a function of osmolytes or denaturants and to study interactions of proteins with polynucleotides, ligands, and other proteins. It is possible to measure the free energy of folding using CD measurements, which is a function of osmolytes or denaturants when the change in CD is caused by the two-state transition of folded and unfolded states. Overall, urea and GuHCl cause a loss of ellipticity ([θ]) but protecting osmolytes (e.g. TMAO, sucrose) shows an increase in ellipticity (88–90).

*Fluorescence spectroscopy*

Fluorescence emission spectroscopy is a biophysical technique widely used in research for analyzing structural conformations and the aggregation characteristics of macromolecules such as proteins. This information is led by studies of a phenomenon affecting the excited state such as the local environment, quenching process and energy transfer. The fluorescence intensity of a molecule is believed to be dependent on its quantum yield obtained by the ratio of emitted photons to that of exciting ones (91). Tyrosine (Tyr) and tryptophan (Trp) known as intrinsic fluorophores get excited at wavelengths around 280 nm, while Trp displays a peak at 295 nm. Their fluorescence properties are sensitive to the environment which changes when a protein folds or unfolds. In native or folded states, Tyr and Trp reside inside the protein core where hydrophobic effect becomes prevalent, giving high quantum yield. While unfolded proteins are exposed to solvents and give rise to the hydrophilic environment (89). There are several on osmolytes (e.g. TMAO, proline, betaine, sarcosine, and sucrose) causing a quantum yield and blue shift in wavelength maxima, suggesting a stable state of proteins in their presence. Studies on mushroom tyrosinase from *Agaricus bisporus *indicated that trehalose as a compatible osmolyte pronounced reduction in maximum emission of intrinsic fluorescence leading to higher stability of the enzyme (93).

*Differential scanning calorimetry (DSC) and isothermal titration calorimetry (ITC)*

Experiments conducted six decades ago indicated that the primary structure of a protein (sequence of amino acids) controls the interactions among component amino acids and as well as between protein and solvent. The results also showed that the interactions were governed by thermodynamics (65–67). There is a delicate thermodynamic balance in proteins; many of them are thermally unfolded at 70 °C and many are denatured by a relatively trivial increase in temperature. While thermophilic proteins stabilized allow organisms to thrive at elevated temperatures and pressures (94). A basic understanding of thermodynamic parameters that leads to the formation of macromolecular noncovalent bonds is achievable using DSC (34). Another calorimetric solution is isothermal titration calorimetry (ITC), which is mostly applied in biophysical studies to measure dynamic events such as kinetic and binding (93). Much effort has been devoted in recent years to understanding the factors responsible for osmolytes stabilizing effect (94). One of the potentially interesting aspects of osmolytes action is their possible effect on the heat capacity change (Δ*C*) and thermodynamic parameters. This aspect is directly interpreted in molecular terms as it mainly reflects the interactions with the solvent of the polar or apolar groups exposed upon denaturation (34, 96). Physical chemists and biologists have no collaboration in the study of osmolytes. That is, why there is not a connection between the molecular mechanisms, thermodynamics, and biological applications of osmolytes. In the past decades, molecular crowding’ hypothesis has been developed to predict the action of osmolytes on protein stability as an excluded volume effect (94–96). This means protein in the native state is smaller than the unfolded state, so a reduction in the space around the protein favors stability and enhances the folding of the structure (95). Studies of protein folding confirm that excluded volume alone does not describe protein folding and a ‘non-specific’ effect was also at play (95). Additionally, it was proposed that protein stabilization was driven by enthalpy not entropy and a mechanism like preferential exclusion (preferential hydration) could be used to describe the experimental observations (97). Thus, hydration of a protein during unfolding experimentally rises its Δ*C*. This is observed when a protein, before being subjected to thermal unfolding in a DSC, is added to compatible osmolyte (e.g. TMAO, betaine, glycine), both the ∆*C*_p_ and *T*_m_ show that ∆*H* of the interior of a protein plays an important role in proteins stabilization (34, 95–97).

*7.4. Sodium dodecyl sulfate-polyacrylamide gel electrophoresis (SDS-PAGE)*

In 1975, O’Farrell introduced two-dimensional PAGE for separating proteins under denaturing conditions that enabled the resolution of hundreds of proteins. In this method, proteins are resolved on a gel using isoelectric focusing (IEF) separating proteins in the first dimension according to their isoelectric point, followed by electrophoresis in a second dimension in the presence of SDS, which separates proteins according to their molecular mass. SDS cleaves non-covalent linked aggregates into monomers, while covalent disulfide bridges remain intact (93). The smallest molecular weight fraction can be attributed to native, intramolecularly cross-linked proteins. Thermal treatment and irradiation lead to cross-linking via the formation of new interactions and an increase of the molecular weight. The behavior of osmolytes against aggregation varies from protein to protein (94). Some osmolytes are found to induce protein aggregation, and others inhibit aggregation of the same protein. For example, glycine and betaine aggregate RNase A while arginine suppresses aggregation. Also, same osmolytes may have distinctive effects on the aggregation of proteins depending upon the structural specificities of proteins, e.g. trehalose, urea, betaine, taurine, and proline. This conflicting role of osmolytes makes it essential to study the effect of each osmolyte on various proteins separately (97–98).

Other analytical techniques such as UV-visible and IR attenuation are not often used for a complete understanding of proteins’ folding/unfolding and investigating protein’s structures and functions (97).

**Table 1:** Chemical instability in proteins.

**Chemical instability**	**Mechanism**	**Proteins**	**Reference**
Deamidation	The hydrolysis of Asparagine (Asn) and Glutamine (Gln)	Human growth hormone (hGH), Insulin, γ-Globulin, Hemoglobin	
Isomerization of Asp	The option cyclic imide intermediates to form either Aspartate (Asp) or iso-Asp products	Monoclonal Antibodies (MAbs)	17–18
Hydrolysis of Asp	Asp-associated hydrolysis of the peptide backbone	Nerve growth factor (NGF)	17, 23
Hinge region hydrolysis	Hydrolysis of the peptide backbone within the hinge region of antibody	MAbs	17–19
Hydrolysis of Trp	Hydrolysis of Tryptophan (Trp) to kynurenine and related substances	Myofibrillar proteins	17, 21
Racemization and β-elimination	Deprotonation of the hydrogen on the α-carbon	Murine lysozyme, IL-1ra, Myelin in muscle	
Diketopiperazine (DKP) formation	Amine attack the second carbonyl group in the peptide backbone and formation of DKP ring	Human growth hormone (hGH)	17, 23
Glycation of Proteins	The reaction with a base, typically the side chain of lysine and a carbonyl group of a reducing sugar	Hemoglobin, Immunoglobulin G2 (IgG2s)	
Formation of pGlu	Nucleophilic attack of the N-terminal amine on the side chain of a Glutamic acid (Glu) residue (and occasionally a Gln residue) to form a five membered ring structure	Bone morphogenetic protein 15 (BMP15)	17–21
Disulfide scrambling	Removal of free Cystine (Cys) residues (the reduced form), which can act as the starting point for disulfide scrambling or exchange	IgG2s	17–18
Oxidations:			
Oxidation of Met	Oxidation of Met accomplished with a wide range of ROS and pH	MAbs	17, 25
Metal-catalyzed oxidation (MCO)	Binding of redox active metal to a protein amino acids (often Gly, Asp, His, and Cys)	Human relaxin, Prolastin, Human growth hormone	17–23
Oxidation of Trp	Oxidation of Trp residue	MAbs	17–21
Photooxidation	Chemical oxidation of light sensitive amino acids e.g. Trp, tyrosine (Tyr), and Phenylalanine (Phe)	MAbs, Milk proteins	17–26
Cysteine Oxidation	Oxidative process involving Cys residues	Alcohol dehydrogenase	17, 26

**Table 2:** Methods for improving of protein stability (24–26).

**Methods**
**Use of aptamers**
**Stabilization by ligand binding to the native state**
Buffers
Surfactant
Cyclodextrins
Anion binding
Polymers
Metal Ions
**Self-assembling**
**Stabilization by drying**
Freeze drying (lyophilization)
Air drying
Vacuum drying
Spray drying
**Artificially modified protein**
Protein chemical modification
Site-directed mutagenesis
Immobilization of protein
Glycosylation
Pegylation
**Conformational stabilization in aqueous solution by excluded solutes (osmolytes)**

**Table 3:** Classification of naturally occurring osmolytes and examples of proteins that stabilized in the presence of osmolytes

**Classification**	**Chemical Name**	**Proteins**	**Reference**
**Methylamines**	Trimethylamine N-oxide (TMAO)	α-Synuclein, Stem bromelain, *Escherichia coli* adenylate kinase, ATPase, Acetylcholinesterase, RNase T1, Lactate dehydrogenase, Protein L, Myoglobin	35–38
	Betaine	Ribonuclease A, Lactate dehydrogenase, Bovine glutamate dehydrogenase, Fatty acid synthase, Phosphorylase b, Myoglobin, Trypsinogen, Lysozyme	38–41
	Choline	Trastuzumab	
**Polyols**	Sucrose	Pea seedling copper amine oxidase, Trypsin, Yeast iso-1-ferricytochrome *c*, Protein L	43–44
	Trehalose	Prion protein, Yeast inorganic pyrophosphatase, β-Lactoglobulin, ATPas, Yeast iso-1-ferricytochrome *c, *Mushroom tyrosinase	45–47
	Glycerol	Human cardiac titin, Yeast inorganic pyrophosphatase, Yeast hexokinase, Rabbit muscle creatine kinase, Insulin	48–49
	2-*O*-α-Mannosyl glycerate (Firoin)	Lysozyme, Prion peptide, β-Amyloid peptide	35, 40
	2-*O*-α-Manno-sylglyceramide (Firoin-A)	Lysozyme, Prion peptide	35, 40
	Mannitol	MAbs, Plasma proteins, Vaccine stabilizer, Factor VIII	35, 50–52
	Sorbitol	MAbs, Plasma proteins, Gonadotropin, Gamma-globulin	53–54
	myo-Inositol	Human thyrocytes	
	Diglycerol phosphate (DGP)	Alcohol dehydrogenase	
	Cyclic-2,3-diphosphoglycerate (cDPG)		
	L,L-Di-myo-1,1´(3,3´)-inositolphosphate (DIP)		
**Amino acids and derivatives**	Proline	Ribonuclease A, Fatty acid synthase, Protein L, Phosphorylase b, Myoglobin, Creatine kinase	35, 57–58
	Glycine	Ribonuclease A, Creatine kinase	59–60
	Ectoine	Trypsinogen, Lysozyme, Prion peptide, Interferon Alfa2b	40, 59, 61–62
	Hydroxyectoine	Trypsinogen, Lysozyme, Prion peptide, Interferon Alfa2b	40, 59, 61–62
	Taurine	Ribonuclease A, Lactate dehydrogenase, Bovine glutamate dehydrogenase,	34, 62
	Serine		
	Gamma-amino-n-butyric acid (GABA)		
	Alanine		
	Sarcosine	Ribonuclease A, Stem bromelain, Pea seedling copper amine oxidase, Barstar, α-Chymotrypsin, Ribonuclease A, anti-Interleukin-6	35, 64
	Citrulline	Urokinase, Peroxidase, anti-Interleukin-6	
	Poly-γ- glutamic acid (L-PGA)		
**Miscellaneous**	Kahalalide F		
	Mycosporine		
	Melanine		
	Bacteriorubin		
	Pannarin		
	Scytonemin		
	Curacin A		
	Dimethylsulfoniopropionate (DMSP)		

## Conclusion and outlook

Based on the years of studies in biochemical and biophysical fields, there has been a great development in the area of experimental and theoretical methodologies to the complex system of proteins, osmolytes, and the surrounding water. Using these findings, researchers of protein science now achieve a deep knowledge in molecular level of these systems. Besides, an attempt has been done to clarify how osmolytes affected on proteins. This fact can be resulted in designing solution media that are more identical to *in-vivo* conditions and make it possible to solve many unknowns related to proteins. Therefore, it is imperative to uncover the behavior of osmolytes towards protein stability. This perspective gives us several osmolytes concerning the stability of diverse proteins. Not all osmolytes’ effects could improve protein stability. Their effect is a function of concentration, presence of other osmolytes, surrounding solvent, condition of solution media (pH, temperature, and pressure), and the nature of a protein among many. Studies on the influence of different concentrations of osmolytes on proteins have led to the introduction of new mechanisms. Doubtlessly, the present perspective is highly important and fruitful to uncover different interactions that support the stability of proteins and the unexpected results with specific osmolytes and proteins. Notably, the stability of a specific protein may not be determined by studies on other proteins. Therefore, this perspective may fuel more studies on proteins that may result in proposing dissimilar strategies for protein stabilization.

## Abbreviations

Asn, Asparagine; Asp, Aspartate; cDPG, Cyclic-2,3-diphosphoglycerate; Cys, Cystine; DSC, Differential scanning calorimetry; DGP, Diglycerol phosphate; DKP, Diketopiperazine; DMSP, Dimethylsulfonoproprionate; Gln, Glutamine; Glu, Glutamic acid; Gly, Glycine; GuHCl, Guanidine hydrochloride; IEF, Isoelectric focusing; ITC, Isothermal titration calorimetry; *T*_m_, Melting temperature; MCO, Metal-catalyzed oxidation; MDS, Molecular dynamic simulation; Phe, Phenylalanine; L-PGA, Poly-γ- glutamic acid; SDS-PAGE, Sodium dodecyl sulfate polyacrylamide gel electrophoresis; TMAO, Trimethylamine N-oxide; Trp, Tryptophan; Tyr, Tyrosine.
